# Hybrid Integrated Label-Free Chemical and Biological Sensors

**DOI:** 10.3390/s140405890

**Published:** 2014-03-26

**Authors:** Simin Mehrabani, Ashley J. Maker, Andrea M. Armani

**Affiliations:** Mork Family Department of Chemical Engineering and Materials Science, University of Southern California, Los Angeles, CA 90089, USA; E-Mails: mehraban@usc.edu (S.M.); maker@usc.edu (A.J.M.)

**Keywords:** sensor, active materials, chemical and biological detection

## Abstract

Label-free sensors based on electrical, mechanical and optical transduction methods have potential applications in numerous areas of society, ranging from healthcare to environmental monitoring. Initial research in the field focused on the development and optimization of various sensor platforms fabricated from a single material system, such as fiber-based optical sensors and silicon nanowire-based electrical sensors. However, more recent research efforts have explored designing sensors fabricated from multiple materials. For example, synthetic materials and/or biomaterials can also be added to the sensor to improve its response toward analytes of interest. By leveraging the properties of the different material systems, these hybrid sensing devices can have significantly improved performance over their single-material counterparts (better sensitivity, specificity, signal to noise, and/or detection limits). This review will briefly discuss some of the methods for creating these multi-material sensor platforms and the advances enabled by this design approach.

## 1. Sensor Background

Over the past few decades, many of the advances in real-time, integrated chemical and biological sensing have been enabled by improvements in fabrication methods or by the increase in computational power for predictive modeling of device performance [1,2]. For example, the ability to create dense arrays of integrated silicon nanowire sensors required the development of nanofabrication techniques and device packaging methods [3]. Although these capabilities were originally motivated by the need for smaller integrated electronic circuits for computers and other electronic devices, they have been leveraged to enable other fields, particularly sensors research. Additionally, advances in computing power have enabled real-time detection and analysis of increasingly complex data signals.

Because integrated sensors can be fabricated in numerous formats, they have impacted nearly every aspect of society, from the automotive industry and defense to healthcare and drug development [4]. As such, the complexity of sensor design varies greatly, from the simple carbon monoxide detector to the gyroscope. However, in all cases, quickly obtaining a reliable and robust signal is critical.

### 1.1. Labeled vs. Label-Free Sensors

**Figure 1.**
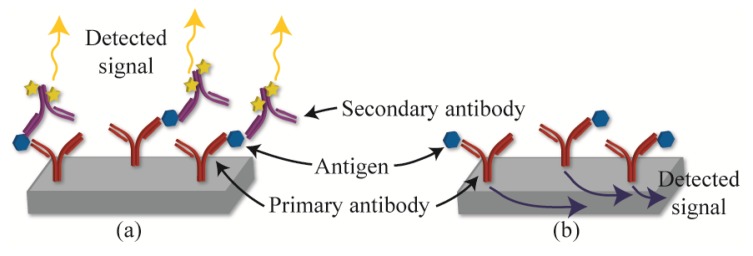
Comparison between (**a**) labeled and (**b**) label-free detection methods.

The present review is focused on label-free detection methods. In contrast to labeled detection, which requires a secondary molecule or amplification step, label-free detection allows the sensors to directly detect the molecule of interest. For example, a labeled detection method (Figure 1a) captures the analyte of interest using primary antibodies, and indirectly detects the bound analyte using fluorescently labeled secondary antibodies. In contrast, a label-free sensor (Figure 1b) is able to directly sense when the protein binds to the antibody. As such, label-free detection can be considered a direct detection modality, and it allows the researchers to detect proteins as they bind in real-time.

One advantage of using labeled detection methods is that the secondary antibody provides dual-confirmation of the presence of the protein, reducing false-positive readings. However, since the secondary antibody introduces an additional time-consuming step, labeled detection methods are not suitable for rapid and real-time sensing applications.

### 1.2. Sensor Overview and Performance Metrics

There are many types of integrated sensors and various approaches for categorizing them. One method is to use the physical transduction mechanism to create classes of integrated sensors. If this method is used, three distinctly different types of sensors are quickly apparent: electrical, mechanical, and optical [1–3,5–8]. An overview of the detection mechanisms and specific examples are shown in Table 1, respectively. However, it is important to note that this table is not meant to be comprehensive, but simply gives the reader a sense of the breadth of research which has been performed in the field. Each sensor was originally demonstrated off-chip, and gradually migrated to an integrated format, also referred to as a Lab-on-Chip. For example, one of the first optical sensors was based on an optical fiber, in which the change between the input power and output power was used as the detection signal [9]. Later, integrated optical sensors were developed using waveguides, resonators, and other on-chip approaches [10–16].

Because of the numerous types of sensors, fundamental metrics were developed for comparing device performance. They are related to the response or behavior of the device. In the present review, we will focus on six of these metrics; however, for the interested reader there are several articles and textbooks which can provide in-depth discussions on sensor theory [4,138].

**Figure 2.**
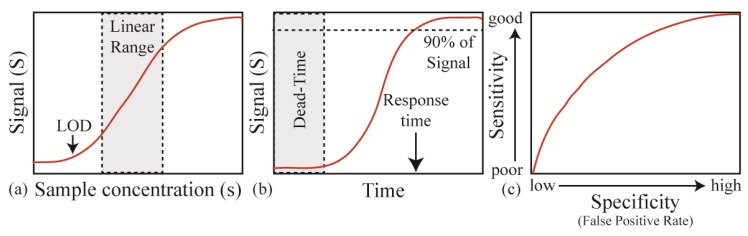
Overview of the key approaches for characterizing sensor performance. (**a**) One approach is to consider the sensor response (S or signal) for a given input or sample concentration (s or sample concentration). This approach would include the linear working range and the limit of detection (LOD); (**b**) Another is to evaluate the temporal behavior of the sensor, which includes parameters such as the response time and the dead-time; (**c**) Finally, the balance between specificity (or selectivity) and sensitivity can be considered. This metric is particularly important in diagnostics.

The key performance metrics include the signal, noise level, signal to noise ratio (SNR), linear range (working range), response time and rate, and false-positive/false-negative rate (selectivity). For clarification, Figure 2 shows an idealized version of a sensor in operation. The signal describes the output signal (S) which is generated with a given input or measurand (Figure 2a). In the linear range of the sensor, this relation is S = a + bs (a = background noise level, b = sensitivity, s = input). Therefore, while a sensor might be able to operate or detect below or above the linear range, because it is out of the linear working range which can be calibrated, these signals will be difficult to quantify accurately.

The signal-to-noise ratio (SNR) value is a critical parameter when considering the suitability of a sensor for real-world applications and is simply the intensity of the signal divided by the noise level. The noise level is the signal with no measurand, and it can vary depending on the environment of operation. The acceptable SNR value will be dependent on the application and on the availability of cross validation methods. For example, a sub-optimal SNR value of 1.5 might be acceptable if multiple sensors are able to provide corroboration of the result.

The limit of detection (LOD) is the smallest measurand concentration which can be reliably detected. This value is typically not included in the working range of a device and can be significantly impacted by noise sources. By improving the SNR, smaller signals can be detected, changing the linear working range and the limit of detection; as such, improving the SNR is of great interest to the sensing community. Depending on the noise source and the sensing mechanism, it is possible to reduce the noise through advanced computational algorithms or the implementation of filters. The development of such techniques is a very active area of research.

The response time and rate describe the temporal behavior of a sensor. Specifically, the response time is the amount of time required for the sensor to reach 90% (typically) of its final value for a given measurand concentration and the response rate is the slope of this curve (Figure 2b). Both the response time and rate of the sensor are governed by the physical mechanism and device properties as well as the measurand delivery method and the signal read-out technique. While advances in sensor design and nanomaterials have significantly improved the response time and rate by increasing the effective sensor surface area, improvements in processor speed have also allowed for increases in response rate and time in sensor systems.

One of the final metrics is the sensor's specificity (or selectivity), which describes how well the sensor specifically detects the analyte of interest. While the previous metrics are related to sensitivity, selectivity is equally important. There are two aspects of selectivity: false-positive rates and false-negative rates. Clearly, the ideal sensor will generate no false-positive or false-negative signals. However, this ideal scenario is extremely unlikely. Therefore, researchers typically design a sensor for a specific application. In other words, for a measurand that has a high probability of harm, it is acceptable to have false-positives. Typically, as shown in Figure 2c, the specificity is directly related to the sensitivity and there is a trade-off in these two metrics.

As sensor design advanced, researchers began to explore combining multiple mechanisms into a single sensor platform to improve the device performance. This approach resulted in two distinctly different paths in sensor design, depending on whether the sensor was designed with the different responses to be generated in parallel or serially. Essentially, the dual mechanism allowed either: (1) a reduction in false-positive signals or (2) an enhancement in the detection signal. For example, one challenge of electrical sensors is a high background noise level due to spurious environmental electric fields. If a mechano-electronic sensor is formed, in which the deformation of a material coating creates an electric signal, the sensor will have a reduced background noise level. On the other hand, the detection signal could be increased if the material coating was also electro-responsive, or changed both its conductivity and its mechanical behavior in response to the stimuli.

As can be inferred from the previous two examples, one of the most straightforward routes of creating hybrid sensor architectures is to combine an active material with an existing high performance sensor. Given the rapid pace of discovery in functional materials [139–141], hybrid sensors are quickly emerging as leaders in this field.

## 2. Integrated Hybrid Sensing Devices

Hybrid sensors are comprised of an underlying sensor device and a secondary layer which operates to improve the performance of the device. Given the numerous key performance parameters described previously, the secondary layer could perform several different functions. For example, it could be an insulating or filtering layer, reducing the background noise and improving the SNR of the device. Alternatively, it could be a biological or chemical targeting layer, improving the selectivity of the device.

### 2.1. Hybrid Sensor Architecture

While there are many routes to design a hybrid sensor structure, one efficient yet simple method is to fabricate the underlying sensor device and then deposit a secondary, functional material on top which can enhance the sensor signal. This approach has two key challenges which are currently the focus of hybrid device research: (1) developing new methods to deposit the active layer and (2) creating novel functional materials. However, it is important to remember that any degradation in device performance will directly impact the sensitivity of the device. Therefore, it is critical to design materials which are optimized for the different device sensing mechanisms.

Due to coefficient of thermal expansion mismatches and fundamental material differences, one of the hurdles in hybrid structure design is delamination between the functional material and the sensor. These issues are similar to those faced by any hetero-material structure, such as heterojunction solar cells [142]. Without uniform contact along the interface, the enhancement offered by the functional material is lost. Because the majority of integrated sensors are fabricated on silicon wafers using conventional lithographic methods, the primary approaches for improving adhesion are surface treatments and tuning the thermal coefficient of the functional material. Given that many of these techniques are fairly well understood and established, the focus of the research is on the creation of novel functional materials and their combination with sensors. As such, the main focus of the present review will be on this aspect of hybrid device research, with a brief discussion of emerging approaches for material deposition methods.

### 2.2. Enhancement Mechanisms

The majority of the functional materials are focused on improving one of the key sensing metrics previously discussed (SNR, selectivity, sensitivity, working range, response time/rate). While the precise functionality of the material is strongly dependent on the sensing mechanism and the target application, the different roles that these materials can play in a larger sensor system are relatively straightforward. For example, to improve the SNR which is related to the sensitivity, one can either increase the signal or reduce the noise. To increase the signal, the material should behave as a low-noise amplifier of the input signal.

Similarly, the conventional route for endowing a sensor with selectivity is to use specific receptors such as antibodies which target specific molecules with high selectivity [143]. Antibodies work on a “lock and key” mechanism, in which the targeted molecule (key) fits precisely into the antibody (lock). Theoretically, while other molecules may bind nonspecifically to the antibody, their lifetime within the binding site will not be as long. However, this mechanism relies on the binding site being in the correct configuration, and antibodies are very sensitive to small changes in temperature or pH and they have a finite lifetime, making storage extremely difficult. Therefore, the development of synthetic molecules which have improved stability and which can replace biologically generated antibodies is a very active area of research [144–149].

## 3. Hybrid Sensing Devices

As mentioned before, based on the physical transduction mechanism, label-free sensors can be classified as optical, mechanical, or electrical sensors. After a brief introduction into each type of sensor, the discussion has been divided into synthetic materials and biomaterials which are applied to the underlying sensor to improve its performance.

### 3.1. Optical Sensors

In integrated optical sensors, chemical or biological detection is based on measuring the change in the refractive index, wavelength, or optical loss of the device as molecules bind within the evanescent field on the device surface [1,2]. Depending on the specific device architecture, this change can be detected in different ways. For example, in a simple optical waveguide operating at one wavelength, the change in the optical loss of the device is a straightforward indicator of the number of bound molecules. However, if a broad band source is used, then the device can operate as an integrated spectrometer.

An alternative, slightly more complex approach is based on resonant devices. Unlike waveguides which have wavelength-independent operation, resonant devices confine light of specific wavelengths. These resonant wavelengths or resonant frequencies are defined by several different parameters of the device, including the geometry and material properties such as refractive index. Therefore when molecules bind to the surface of the device, the resonant wavelength changes. Because the detection signal in a resonant device is based on both the geometry and the refractive index, deformable hybrid materials offer a straightforward way to enhance the detection signal [134].

#### 3.1.1. Synthetic Materials

Combining functional polymeric materials with integrated optical sensors has enabled improvements to biological and chemical detection as well as environmental monitoring. One reason for this impact is the wide range of low optical loss, yet highly responsive polymeric materials which can be easily combined with optical devices. For example, both polystyrene and polymethylmethacrylate have extremely low optical loss (high transparency) at a wide range of wavelengths and are easy to synthesize and deposit on silica and silicon devices. Because of the simple backbone of the polymer, the inclusion of functional or responsive groups is straightforward. Additionally, the low optical losses of the polymers ensure the sensor's performance is not significantly degraded by the polymer materials.

Attachment of responsive polymer layers, namely carbohydrate sensitive hydrogels, to optical sensors has proved especially beneficial to glucose sensing. The first demonstration of this device had a dip-coated hydrogel layer on a fiber-embedded Fabry-Perot cavity [150]. As deposition methods advanced, researchers transitioned to spin-coating on planar optical devices, enabling the creation of integrated structures. However, these devices still relied on the dye molecule to enhance the detection signal above the baseline noise level, which limited the ultimate lifetime of the devices. Therefore, advances in both sensor design and functional polymers were needed.

**Figure 3.**
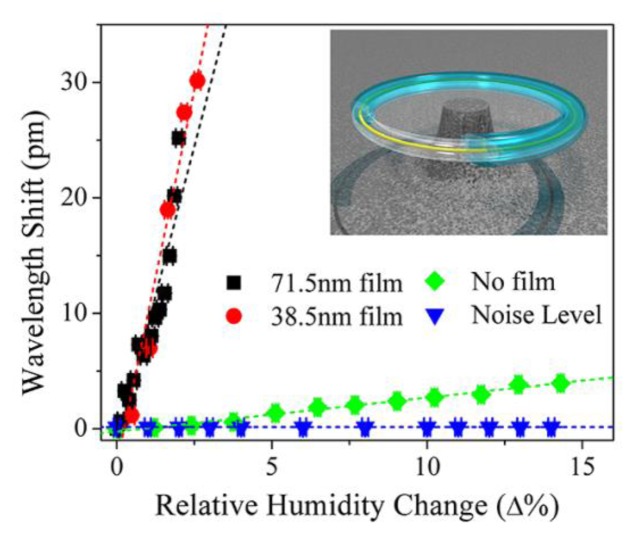
Resonant wavelength shift as a function of changes in the relative humidity at 23 °C for bare silica microtoroid, and pNIPAAm coated silica microtoroids (two different film thicknesses) as well as the noise level measurements. Addition of the pNIPAAm polymer coating improves the response of the bare silica device by nearly two orders of magnitude. The inset is a rendering image of the hybrid polymer coated silica microtoroid. Adapted with permission from reference [134] ^©^ 2013 American Physical Society.

Recently, advances in both fields have finally converged to create a solution to this challenge through integration of silica optical mircroresonators and poly (N-isopropylacrylamide, pNIPAAm). pNIPAAm was deposited on silica microtoridal resonators using the initiated chemical vapor deposition (iCVD) technique. As the relative humidity changed, the resonant wavelength of the hybrid device shifted due to the induced change in both refractive index and the size of the polymer coating. As can be seen in Figure 3, this polymer coating increased the response of the bare silica device by nearly two orders of magnitude [134].

In addition to humidity-responsive polymers, other polymers have been developed which are selective to different gases [151]. Karakouz *et al.* [152] used polystyrene (PS) and polystyrene sulfonic acid (PSS) in combination with localized surface plasmon resonance sensors to detect chloroform, water vapor, toluene and methanol. These polymers swell or shrink after exposure to different gases which change the local refractive index, inducing a shift in the localized surface plasmon resonance peak. Poly (methyl methacrylate) (PMMA) has also been applied in surface plasmon resonance detection of gas molecules. Ma *et al.* [153] applied a thin layer of PMMA on silver nanoprisms and used this platform for the detection of chloroform. Upon exposure to this gas, PMMA film swells and not only increases the response of the sensor, but also acts as a selective layer to chloroform in the presence of six other vapors. PMMA and PS are both ideal polymers for optical devices because they have very low inherent optical loss and as such do not degrade the underlying device performance.

Metal-organic frameworks (MOFs) are another group of synthetic materials that have been recently introduced into the field of chemical detection. MOFs are highly porous materials with a great degree of tunability in their structural, chemical and physical properties. In addition, unlike most polymeric materials, MOFs are stable even at very high temperatures of up to 300 °C [154,155].

**Figure 4.**
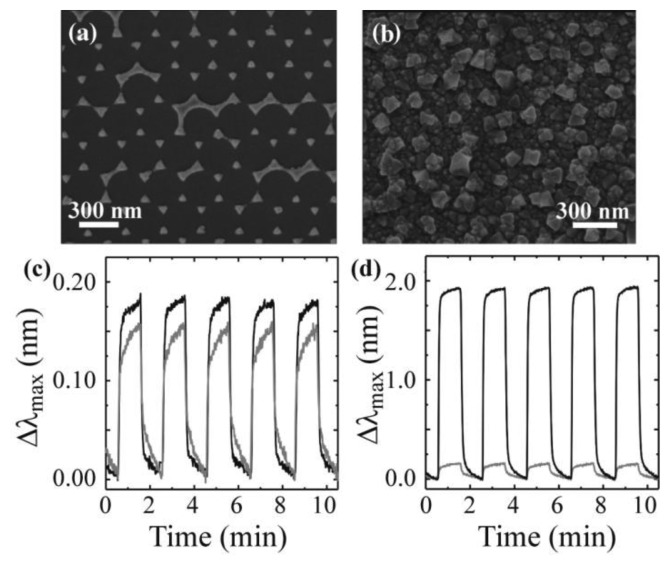
SEM images of (**a**) a triangular silver nanoparticle array fabricated by nanosphere lithography on a glass coverslip and (**b**) silver nanoparticle array coated with benzenetricarboxylate forming the MOF sensor platform; (**c**) Response of the bare nanoparticle sensor to SF_6_ (black) and CO_2_ (gray); (**d**) Response of the bare nanoparticle (gray) and MOF-coated nanoparticle (black) to CO_2_. The coating increases the response by approximately an order of magnitude. Adapted with permission from reference [129] ^©^ 2010 American Chemical Society.

Recently, Kreno *et al.* [129] combined MOFs with a localized surface plasmon resonance sensor based on silver nanoparticles to demonstrate the detection of CO_2_ gas molecules. The addition of a MOF material (Cu_3_(BTC)_2_(H_2_O)_3_, BTC = benzenetricarboxylate) on the silver nanoparticles amplified the response by 14-fold (Figure 4). Additionally, as can be observed, the surface could be quickly recycled, creating a reusable device.

#### 3.1.2. Biomaterials

Recent efforts to improve biological and chemical sensors have also led to the development of novel sensors made with biomaterials. Fabricating optical sensors using biomaterials has several important advantages and limitations. First, biomaterials are inherently biocompatible. As a result, adding biomaterials to sensors can help stabilize biomolecules and improve the accuracy and efficiency of detection. Many biomaterials are also transparent, enabling optical sensors to be coated or fabricated entirely from them to increase biocompatibility and sensitivity [156–159]. For example, transparent biopolymers, lipids, and hydrogels can be easily added to optical sensors to increase biocompatibility without significantly compromising optical performance [160–162]. Some materials such as agarose hydrogels [160,163] and *E. coli* cells [164] can be made into integrated optical devices. For example, by controlling the amount of crosslinking, the resulting optical and material properties and thermal stability can be tuned [161]. By changing the agarose concentration, the light can be confined inside waveguides with a 2 wt.% agarose core and 1.5 wt.% agarose substrate. Additionally, live cells could be encapsulated inside these agarose waveguides and the entire waveguide device could be integrated with microfluidic channels [163]. Another promising biomaterial is silk fibroin, a protein which is robust and has high transparency [165]. Because of its favorable optical properties, silk has been used to create optical materials such as lenses and gratings by nanoimprinting or molding [166] and it can also be used as a flexible, biodegradable substrate [167]. Improving biocompatibility with these materials will allow optical-based monitoring systems *in vivo* and *in vitro* with higher stability.

A second advantage of using biomaterials in optical sensors is the ability of many biomolecules to bind analytes strongly and with high specificity, even in complex environments [8,113,168]. Many biomolecules are known to have strong affinity for or sensitivity to certain analytes or stimuli. While antibodies are very commonly used to probe for antigens of interest, there are numerous other possible bio-interactions which can be leveraged to create sensor platforms [114,118,169–171]. For example, thymine-thymine base pairs on DNA can strongly bind to mercury ions [172] and hemoglobin can probe for oxygen, CO_2_, and CO, as well as cyanide [116]. By covalently attaching these biomolecules, including antibodies and receptor proteins, to the device surface, it is possible to specifically target a given analyte. Numerous methods have been developed to covalently attach biomolecules to silica [123,171], silicon [18,123], and noble metals [126,127] improving specificity. Developing new surface chemistries for optical sensors is crucial in order to achieve specific and sensitive detection. By optimizing the surface functionalization procedures, optical sensors can be developed which are capable of detecting single molecules, cells, and nanoparticles [15,120,173,174]. Effective detection of analytes is especially crucial in medical and diagnostic applications.

**Figure 5.**
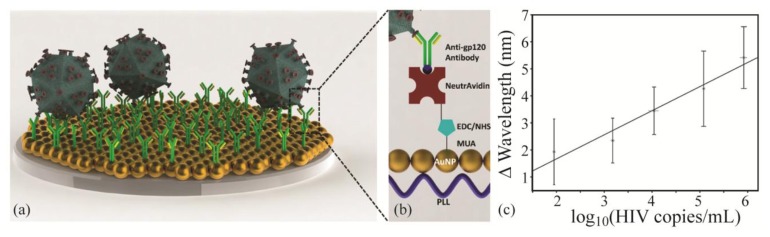
(**a**,**b**) Schematics showing attachment of antibodies to gold nanoparticles to target specific HIV subtypes using antibodies; (**c**) By monitoring shifts in the excitation peak wavelength, HIV virus subtypeB can be detected from whole blood samples. Adapted with permission from reference [113]^©^ 2013 American Chemical Society.

Recently, a plasmonic optical sensor based on gold particles and antibodies was functionalized with various antibodies specific to human immunodeficiency viruses (HIV). Bovine serum albumin was also attached to the sensor surface as a passivation layer to help block nonspecific binding. As a result of this effective surface functionalization, various subtypes of HIV could be detected in whole blood with high sensitivity and repeatability (Figure 5) [113]. The ability to rapidly diagnose the presence of HIV could significantly improve treatment and patient care, particularly in places where access to medical care is difficult and the probability of repeated patient visits is low.

Another interesting approach is to develop hybrid optical materials which combine both biomaterials and optical materials. Proteins sensitive to specific analytes, temperature, and pH can be incorporated into optical and biological materials such as hydrogels, silicone [175] and sol-gel silica [128,135,176–178]. For example, living E. coli have been successfully encapsulated in sol-gel silica by Rajan *et al.* [135]. Various strains of E. coli were genetically engineered so that they would exhibit luminescence in response to stressors such as heat shock and peroxides. By monitoring the luminescence in the E. coli, the stress level of the bacteria could be tracked. Since the bacteria remained viable for several months, these luminescence sensors could be used as early warning sensors. As seen in these examples, incorporating biomaterials into optical sensors can significantly improve the specificity and selectivity of detection.

As outlined in the previous sections, combining biomaterials with optical sensors can significantly improve sensitivity, specificity, and biocompatibility. Biomaterial-based optical sensors can also be significantly more biodegradable and environmentally friendly compared to other types of optical sensors. This can make the fabrication and disposal of the optical devices much safer and potentially less expensive. Low-cost and disposable biosensors are especially desirable in medical diagnostic applications [119,179]. Additionally, sensors made from biocompatible and biodegradable materials could find applications *in vivo* as implantable biosensing devices [180,181].

However, it is important to acknowledge that biomaterials also introduce some important limitations. First, biomaterials are fragile and generally cannot withstand extreme conditions such as high temperature and pressure, strong chemicals or extreme pH. As a result, biomaterials must be handled and stored carefully. Additionally, biomaterials are generally not compatible with the harsh chemical etchants and microfabrication processes used to make traditional optical sensors. To mitigate this issue, biomaterials are typically applied to completed or nearly completed devices. New advances in fabrication processes, such as soft lithography [130,165,182], dip-pen lithography [122], inkjet printing [157,183], and development of ultraviolet-patternable biomaterials [157,182] will further enable fabrication of improved optical sensors with biomaterials.

#### 3.1.3. Additional Considerations for Hybrid Optical Sensors

As seen in the previous sections, adding synthetic or biological materials to optical sensors can significantly improve device performance. In most applications, the performance of optical sensors is ultimately limited by the sensor's optical properties, including the refractive index and absorption coefficient. For hybrid sensors, it is therefore important to implement synthetic and bio-based materials which have controllable refractive index and are highly transparent in order to maintain sensor's performance. Some materials such as polymers can have nonlinear optical properties which need to be considered as well. For example, if the refractive index of a hybrid coating is different than the refractive index of the underlying optical sensor, the optical modes and behavior of light in the sensor could change and alter the sensing performance. Similarly, a hybrid material which has high absorption loss could degrade the sensor performance.

In addition to the optical properties, it is also important to consider the compatibility of hybrid materials and coatings with the optical sensors' fabrication and operation conditions. For example, some materials such as polymers have favorable optical properties for hybrid sensors, but are not compatible with existing fabrication approaches. Often it is necessary to apply the hybrid materials near the end of the fabrication process using spin-coating, dip-coating, vapor deposition, sputtering, surface chemistry, or other methods. These processes must be carefully optimized to uniformly and successfully apply the hybrid materials while preserving the sensor's performance, maintaining the favorable properties of the hybrid materials, and ensuring the hybrid materials are stable and will not delaminate or crack on the sensor surface.

### 3.2. Mechanical Sensors

The working principle of mechanical sensors is based on a change in the mechanical characteristics of the transducer as a result of a chemical or biological stimulus [74,85,90]. Cantilever sensors, emerging in the mid 1990's [62], are the most common type of mechanical sensors and are typically made of silicon/silicon oxide [68,76,93,184], silicon nitride [79,185–187] or polymeric materials [65,188,189]. Cantilevers have two main working modes: static mode and dynamic mode.

In the static mode or deflection mode, the binding of a chemical or biological species only occurs on one side of the cantilever which results in an unbalanced surface stress. This causes the cantilever to deflect up or down [190]. The equation that relates the cantilever displacement to the surface stress is based on Stoney's equation:

(1) $$z_{\max}=\frac{3l^{2}\left(l-v\right)}{Et^{2}}\Delta \sigma$$

where z_max_ is the cantilever's displacement, *l* is the cantilever's effective length, *v* is the cantilever's Poisson ratio, *E* is the cantilever's Young's modulus, *t* is the thickness of the cantilever and σ is the differential surface stress induced as a result of the binding of the chemical or biological species on the cantilever. As such, the sensitivity of the device can be tuned by modifying the Young's modulus of the material, as well as length and thickness of the cantilever.

The second operational mode is the dynamic mode or the resonant mode. Any micro-cantilever vibrates at a certain resonant frequency which is described using a spring-mass system through the following equation:

(2) $$f_{o}=\frac{1}{2\pi }\sqrt{\frac{k}{m^{*}+\alpha \Delta m}}$$

where *f_o_* is the resonant frequency, *k* is the spring constant of the cantilever, m* is the effective mass of the cantilever which is dependent on the environment, *Δm* is the change in mass due to analyte binding, and *α* is a correction factor to account for the location along the cantilever where the analyte binds. Similar to the static mode, the sensitivity (*Δm*) can be tuned by changing the material properties and the cantilever geometry. However, there is also an additional dependence on the viscosity of the environment, which appears in the expanded form of the m* term.

Specifically, cantilevers working in the static mode are usually longer and softer compared to the dynamic mode and are more commonly used for applications in aqueous environments because their response is not impacted by the high viscosity of water [191]. On the other hand, when operating in ideal environments, dynamic mode cantilevers are capable of detecting smaller changes than the mass of the cantilever [85]. Recently, a cantilever with an embedded microfluidic circuit was developed [192]. This approach combines the best of both methods, as it enables detection in aqueous environments using the high sensitivity dynamic approach while isolating the cantilever from the high viscosity environment. With the advent of this method, single protein counting in realistic environments has been achieved.

In the early studies on cantilevers, especially for the static mode sensing, the cantilevers were typically coated with inorganic coatings such as gold [101,188,193], palladium [59], and platinum oxides [63,194]. However, these inorganic materials suffer from lack of selectivity for specific chemical or biological analytes; therefore, it is necessary to apply organic materials on the surface of the cantilever sensors to not only realize selective detection but also to improve their performance metrics.

#### 3.2.1. Synthetic Materials

**Figure 6.**
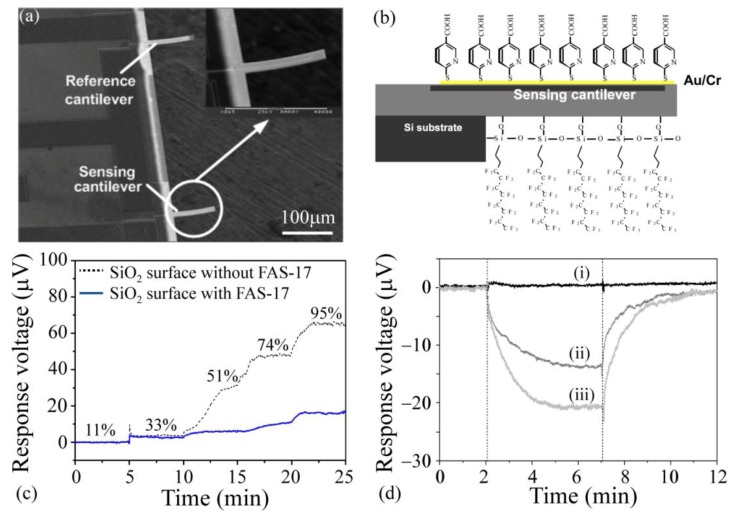
(**a**) Scanning electron micrograph of the cantilever; (**b**) Schematic of the cantilever cross section which shows the dual-SAM modification on two sides of the cantilever; (**c**) Response of the sensor with and without the hydrophobic modification of FAS-17 SAM on the non-sensing side toward different levels of humidity; (**d**) The effect of various SAM modifications on the cantilever sensing response: (i) cantilever without SAM; (ii) cantilever with 4-MBA SAM; (iii) cantilever with 6-MNA SAM. Adapted with permission from reference [111] ^©^ 2007 IOP Publishing Ltd.

One of the interesting synthetic materials used for imparting selectivity and sensitivity to microcantilever sensors is self-assembled layers of organic materials [101,195,196]. The high rigidity and specific orientation of self-assembled monolayers (SAMs) provides a robust platform for adding functional groups while helping with the elimination of dimerization and intermolecular bonding. The receptor functionalized on the SAMs depends on the type of the target analytes. Previous work has used SAMs of 4-mercaptobenzoic acid (4-MBA) and 6-mercaptonicotinic acid (6-MNA) on gold coated silica cantilevers for detection of various explosive vapors such as pentaerythritol tetranitrate (PETN), hexahydro-1,3,5-triazine (RDX) and trinitrotoluene (TNT). The 6-MNA modified cantilevers demonstrated superior selectivity toward TNT mainly due to the size matching between the benzene-ring in the 6-MNA sensing layer and the TNT molecule (Figure 6) [94,95,111,197]. Another interesting application of SAMs is to coat a hydrophobic SAM on the non-sensing side of the cantilevers to block the adsorption of moisture and oil vapors from the environment, thereby reducing false-positives and reducing the background noise. Zuo *et al.* [111] coated the non-sensing side with heptadecafluorodecyltrimethoxysilane (FAS-17) SAM which resulted in about 20% improvement in the suppression of the environmental effects (Figure 6).

In addition to applying SAMs to modify the surface of cantilevers, other types of polymeric coatings have been studied to improve the performance of these sensors [55,60,67,91,104,109,198–203]. Interestingly, not only the type of the polymer coating but also the location of the coating on the cantilever affects the performance of the device [204]. Pinnaduwage *et al.* [96] used a silicon based polymer, SXFA-[poly(1-(4-hydroxy-4-trifluoromethyl-5,5,5-trifluoro)pent-1-enyl)methylsiloxane], on the sensing side of the cantilever for the detection of 2,4-dinitrotoluene (DNT). One of the important characteristics of this sensor is its long-term stability which is mainly due to the fluorocarbon groups in the polymer coating. The C-F bonds are thermodynamically stable and the bulky CF_3_ groups protect the polymer backbone through steric hindrance.

Polymeric materials blended with metals are also applied on the cantilevers where the metal atoms act as a catalyst for the reaction of the target molecule with the polymer film. Kooser *et al.* [82] used a composite of poly(ethylene oxide) and nickel as the functional layer for CO sensing. Macrocyclic oligomers with cavities of molecular dimensions such as *tert*-butylcalix[6]arene (TBC6A) are also used as selective coatings for the detection of trinitrotoluene (TNT) vapors. In these coatings, the basket-like molecular cavities help with the increase in the affinity of the analyte to the sensor surface [205]. Polymers that allow for the sorption of analytes in aqueous environment are also of interest in microcantilever sensing. Poly(isobutylene) (PIB) and poly(ethylene-co-propylene) (EPCO) have been successfully used for the detection of various toxic and carcinogenic contaminants in water [61].

One of the drawbacks of polymeric coatings is their slow recovery as well as degradation over time when compared to the underlying device [206]. To overcome these issues, metal organic frameworks (MOFs) have been recently suggested. The high porosity and surface area of these materials enhances the diffusion of chemical species into and out of the coating, yielding higher sensitivity and faster response time. Microcantilevers modified with MOFs composed of benzenetricarboxylate ligands that link Cu (II) ions were used by Allendorf *et al.* [54] for the detection of various vapors such as methanol, ethanol and water. Their studies revealed that the diffusion of these vapors in the MOF film changes the crystal structure which translates into a change in the mechanical properties of the hybrid cantilever sensor. Additionally, modeling of the electromechanical behavior of the MOF coated cantilevers showed the effect of different mechanical properties of MOFs on the sensor response and the potential of using MOFs for the selective detection of nerve agents, explosives, and toxic chemicals [87,207].

#### 3.2.2. Biomaterials

When combined with bio-based recognition agents, cantilevers have been widely applied for the detection and characterization of various biological species such as DNA, RNA, cytokines, peptides, disease biomarkers, bacteria, viruses, and spores [64,208]. The primary type of bio-recognition layer consists of naturally derived receptors that have high affinity and selectivity for the target biomolecule [209]. Examples of these receptors include antibodies [57,66,71,75,76,88,89,98,99,102,105,108,210–220], nucleic acids and aptamers [70,77,80,81,86,100,103,106,110,221–226], peptide ligands [72,84,92,227], and phages [73,228]. The antibodies have carboxyl and amine groups; therefore they can be immobilized on the silicon-based cantilevers through silanization of silicon. In the case of gold coated cantilevers, the self-assembly of thiolated receptors can be used [229]. In addition, nano-assembly layer-by-layer (LBL) deposited biocompatible polymer films can serve as the platform for the attachment of the non-thiolated receptors through chemical reaction or electrostatic forces [230].

Various biomaterials have been studied for the detection of glucose using hybrid cantilever sensors. The enzyme glucose oxidase (GOx) was immobilized on the surface of gold coated cantilevers modified with poly-L-lysine using glutaraldehyde (GA). The enzyme reaction between glucose and GOx results in bending of the cantilever [107]. GOx demonstrates high selectivity toward glucose detection in the presence of other sugars such as D-fructose and D-mannose [231]. GOx has also been immobilized on cantilevers using layer-by-layer nanoassembly of cationic polyethyleneimine (PEI), and anionic poly(sulfonate styrene) (PSS) [232]. In this case, the sensor demonstrated selectivity for glucose in the presence of other monosaccharaides, such as mannose, fructose, and galactose [233]. In addition to glucose oxidase, biocompatible glucose responsive polymers have been applied as coatings on cantilevers for glucose detection. These polymers include poly(N-isopropylacrylamide)-copoly(acrylic acid)-(3-aminophenylboronic acid) (PNIPAAM-co-PAA-PBA) [69] and poly(acrylamideran-3-acrylamidophenylboronic acid) (PAA-ran-PAAPBA) [234].

There are two challenges in the detection of biomolecules using bio-recognition receptors with cantilevers: (1) binding of non-target species in the presence of other proteins and bio species *i.e.*, in whole blood or human serum and (2) the requirement that the bio-agent is stabilized in an aqueous environment.

**Figure 7.**
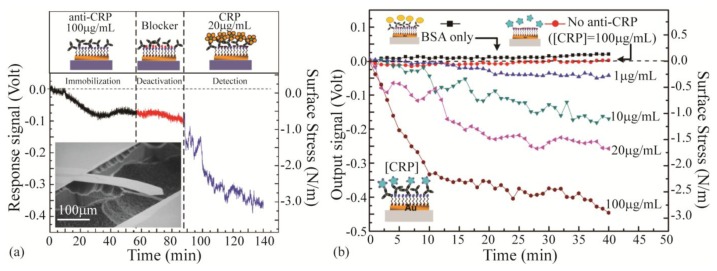
(**a**) Behavior of gold coated cantilever toward immobilization of the anti-CRP as the recognition agent, and ethanolamine as the blocking agent as well as the detection of analyte of interest: CRP. Inset: Scanning electron micrograph of the cantilever; (**b**) Response of the sensor toward different levels of CRP along with control experiments on bovine serum albumin (BSA) which shows the high specificity of the sensor toward CRP. Adapted with permission from reference [238] ^©^ 2013 by the authors; licensee MDPI, Basel, Switzerland.

Nonspecific binding can cause false positive results which affect the selectivity and the sensitivity of the sensor. In order to overcome this problem, passivation layers are used to block nonspecific binding on the sensor. The passivation layer is composed of small inert biocompatible molecules that fill in the voids of the sensor surface as well as its non-sensing side. Bovine serum albumin (BSA) is one blocking biolayer that has been used in microcantilever biosensors [78,92,235]. Casein is another blocking agent applied in cantilever biosensors which shows superior blocking compared to BSA [75]. In addition, polyethylene-glycol (PEG) has been used as a passivation layer [97,105,236]. PEG is a protein-resistant and anti-fouling polymer with low toxicity and high biocompatibility [58]. Studies on cantilevers with both BSA and PEG layers revealed that PEG is more successful in depression of nonspecific binding [83,237]. Yen *et al.* [238] recently demonstrated the application of ethanolamine as the blocking agent in the detection of C-reactive protein (CRP). They immobilized the anti-CRP as the recognition layer on gold coated cantilever through self-assembled monolayer of 8-mercaptooctanoic. The ethanolamine was then used to block the voids in the self-assembled monolayer. Figure 7a depicts the effect of each of these treatments on the sensor surface as well as the response of the sensor toward CRP. Figure 7b demonstrates the response of the device toward various concentrations of CRP.

#### 3.2.3. Additional Considerations for Hybrid Mechanical Sensors

The development of materials for hybrid mechanical sensors is inherently difficult. Many of the properties which improve specific sensor metrics result in the degradation of the overall device. For example, a highly elastic functional material will enable a faster sensor response; however, due to a mismatch in material expansion coefficients, the probability of delamination between the functional material coating and the underlying sensor is high.

Another important issue to consider is the hybrid materials' compatibility with the fabrication processes used to make mechanical sensors. Some materials such as polymers and biomaterials cannot tolerate high temperatures and harsh chemical environments, and therefore need to be added at the end of the fabrication process using surface chemistry, vapor deposition, or other methods. Given that many mechanical sensors are suspended structures, such as cantilevers, these deposition processes can be rather complex.

### 3.3. Electrical Sensors

The first electrical sensors were rooted in Ohm's Law (V = IR) and were based on detecting a change in one of the three variables (voltage, current or resistance) as analytes bound to the surface of the device. As time progressed, additional approaches for electrical detection were developed, including methods based on capacitance and electrical potential [239–248]. However, because these methods relied on directly measuring linear response in the electrical sensor, they were highly susceptible to noise.

An alternative method based on measuring the first derivative of this signal is a field effect transistor (FET) sensor. In addition to higher sensitivity, FET sensors also have a faster response [47,249]. These devices are mainly composed of a semiconductor (e.g., p-type silicon) with two diffusion regions (e.g., n-type silicon) as the source and drain, which is covered with an insulating layer (e.g., silicon dioxide) and a gate electrode (e.g., palladium) on top. Applying a positive voltage to the gate electrode (V_g_) builds an electric field perpendicular to the surface of the semiconductor, causing holes to move close to the semiconductor surface forming a depletion region. Once there is a voltage difference between drain and source (V_d_), electrons move along the depletion region and form a conduction channel. The conductivity of this channel or the magnitude of the source-drain current (I_d_) depends on the magnitude of the electric field perpendicular to the surface of the semiconductor. Therefore, if a chemical or biological species binds on the surface of the gate and changes this electric field, it can be detected through monitoring the changes in the source-drain current (I_d_). Gate electrodes are usually made of thin metal films of palladium, platinum or iridium. In the case of high temperatures (600 °C–800 °C), silicon carbide or superconducting cuprate can be used [250].

#### 3.3.1. Synthetic Materials

One of the interesting synthetic materials used in electronic sensors are conducting polymers (CPs) [251]. Originally developed for applications in solar cells and electronics, these materials provide the electrical properties of conducting materials and at the same time, they offer the advantages of polymeric materials. Specifically, they are relatively easy to be deposited and they have good mechanical properties which facilitate their application in sensors [17]. The electrical properties of CPs originate from their π-conjugated backbones. Through injection of holes or electrons (doping) into the conjugated backbone, a self-localized electronic state forms in the previously forbidden semiconductor bandgap which transforms the material into a conducting state [252]. CPs such as polythiophene (PT) are stable without the addition of any dopants, however, most CPs are only stable in the form of a p-type semiconductor; for example, polyaniline (PANI), polypyrrole (PP) and poly(phenylene sulphide-phenyleneamine) (PPSA) [32].

One of the applications of CPs is in field-effect transistor (FET) sensors where a thin film of the conducting polymer is deposited on silicon substrate to form the gate of the FET. The interaction of the analyte of interest with the polymeric gate material affects the work function of the system which forms the basis of detection in these sensors [47,252]. Various CPs have been used as the gate material in FET sensors for the detection of different gas molecules. Examples include polypyrrole [253–265], poly- and oligo-thiophenes [27,42,256–266], pentacene [34,50,259,267,268], phthalocyanines (metallophthalocyanines) [21–23,269,270], carbon black composite polymers (poly(ethylene-co-vinyl acetate), poly(styrene-co-butadiene) and poly (9-vinylcarbazole)) [271], along with poly(ethylene-co-vinyl acetate) (PECVA), poly(styrene-co-butadiene) (PSB), and poly(9-vinylcarbazole) (PVC) [26]. FET sensors with conductive polymeric gates are also used for the detection of chemical species in aqueous environments. In order to make the device compatible with an aqueous environment, different approaches have been applied. Hydrophobic fluorinated polymer coatings have been used to protect the non-sensing areas of the device. In this case, the choice of the gate material is limited to water compatible CPs such as pentacene, R-sexithiophene (R6T), dihexyl R6T (DHR6T), and copper phthalocyanine (CuPc) [272]. In addition to hydrophobic coatings, poly(dimethylsiloxane) (PDMS) microfluidic channels have also been used to carry the fluid to the pentacene gate [40].

Another class of newly developed electronic sensors is based on carbon nanotube field-effect transistors [5,44,46,51,69]. The adsorption of molecules on the structure of SWNTs (single-wall nanotubes) which is only made of carbon surface atoms causes drastic changes in their electronic properties [45]. These changes are measured through monitoring conductance or capacitance of the SWNTs which are typically the gate material in the field-effect transistor sensing structure [46]. The first demonstration of sensing gas molecules using SWNTs was performed by Kong *et al.* [36] where the SWNTs formed a p-type transistor and were used for the detection of the NO_2_ gas molecules (electron acceptor). After the successful demonstration of the applicability of carbon nanotubes for gas sensing, various studies focused on improving the sensing ability of carbon nanotubes. One of the most successful demonstrations is carbon nanotube-polymer composite sensors [33,37,39,273,274]. The covalent attachment of poly (m-aminobenzene sulfonic acid) (PABS) to SWNTs improved the selective detection of NH_3_ gas molecules. In this case, the PABS gets deprotonated through exposure to the NH_3_ gas molecules which decreases the conductance of the hybrid device due to the covalent bond between PABS and SWNTs [19]. Another polymeric material used to increase the sensitivity of the SWNTs is polyaniline (PANI) [38]. In this approach, the interaction of the polyaniline with the SWNT increases the π-electron delocalization that results in an increase in the charge transfer between the PANI film and the nanotube [275].

**Figure 8.**
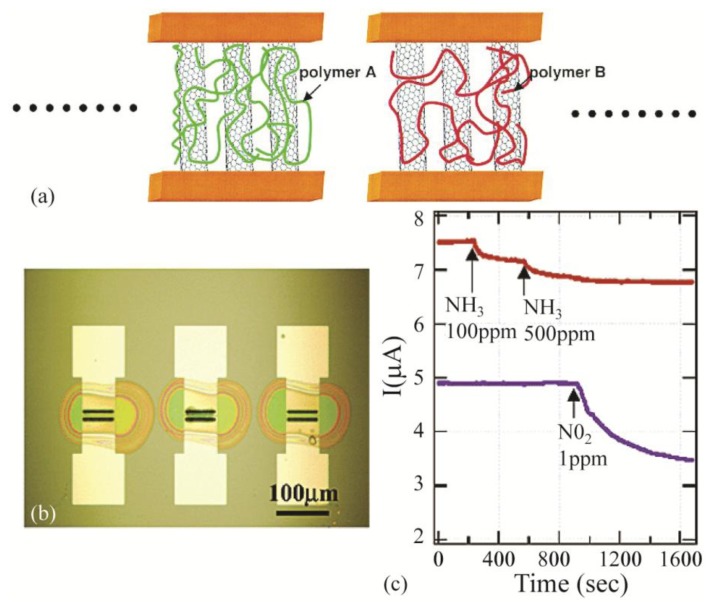
(**a**) Schematic of SWNT sensor. In the present work, Nafion and PEI are the polymers used; (**b**) Optical image of three sensors after coating with droplets of polymer solutions; (**c**) Comparison between the Nafion coated and the PEI-coated devices: The red (top) curve is the response of a Nafion coated device which shows response to 100 ppm and 500 ppm of NH_3_ in air and no response when 1 ppm NO_2_ was injected. The blue (bottom) curve is the response of a PEI-coated device which shows no response to 100 ppm and 500 ppm of NH_3_ and a large drop in conductance to 1 ppm of injected NO_2_. Adapted with permission from reference [276] ^©^ 2003 American Chemical Society.

Polymeric films also improve the selectivity of the carbon nanotubes. Qi *et al.* [276] studied two different polymer coatings of polyethyleneimine (PEI) and Nafion. The PEI coating demonstrated selectivity toward the NO_2_ molecules in the presence of other gases. In addition, it improved the NO_2_ detection limit compared to bare SWNTs. The main reason for the increase in the detection sensitivity is due to the electron-rich nature of the PEI coating. On the other hand, the Nafion coating increased the sensitivity and selectivity of the sensor toward the NH_3_ gas molecules. This is mainly due to perm-selectivity of sulfonic acid side groups in Nafion to –OH-containing molecules like NH_3_. Figure 8 demonstrates the sensitive and selective response of the device with two different polymer coatings of PEI and Nafion.

#### 3.3.2. Biomaterials

Numerous advances in biomaterials have significantly improved electrical sensors as well. Since some conductive materials, like carbon nanotubes, are known to have cytotoxic effects, combining them with biomaterials can improve the biocompatibility and stability of sensing devices [277–279]. Also, attaching biomaterial and passivation layers to the surface of electrical sensors has enabled these sensors to achieve label-free detection as well as significantly higher sensitivities and selectivities compared to unmodified devices. As a result, biomaterial-functionalized electrical sensors have been demonstrated capable of detecting even single molecules.

As with optical and mechanical sensors, attaching biomaterials to electrical sensors has enabled specific and label-free detection of analytes. Antibodies, DNA, and other receptors can be attached to probe for specific analytes of interest. In addition, some biomolecules can probe for other stimuli, such as bacteriorhodopsin's sensitivity to X-rays [280]. Various approaches have been implemented to attach these biomaterials to carbon nanotubes, metal nanowires, and other electrical materials without degrading sensor's performance [281]. Carbon nanotubes are commonly oxidized to produce carboxyl groups, which are reacted with amine-rich biomolecules via EDAC (N-ethyl-N-(3-dimethylaminopropyl) carbodiimide hydrochloride) [19,28,125]. Metals such as gold are frequently functionalized using thiol chemistry [282]. Using these methods, electrical sensors based on nanopores [20], nanowires [3,35,49,53,283,284] and carbon nanotubes [25,28,33,36,45,125,275,279] have been developed which can selectively detect low concentrations of analytes and even single molecules and viruses [20,43,283].

Nanopore-based sensing methods have especially benefited from this surface functionalization approach. As an electric field pulls molecules such as DNA through nanopores, the current changes, and the size of the molecules can be deduced based on the duration of the current change [285]. Initially, nanopores could detect molecules of different sizes or lengths, but could not distinguish between similar molecules [285,286]. By attaching specific adaptor molecules to the nanopores, implementing naturally occurring biological nanopores, and/or using hybrid synthetic nanopores, it is possible to detect specific biomolecules and use nanopores in applications such as label-free DNA sequencing [20,29,48,285,287]. These approaches may enable rapid and inexpensive detection useful in many applications, including affordable sequencing of the human genome.

**Figure 9.**
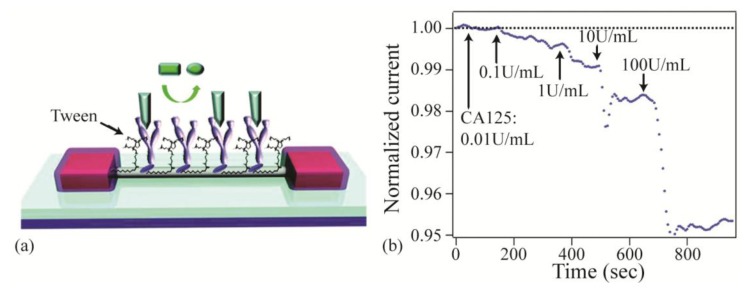
(**a**) Schematic of nanowire sensing platform, showing the Tween20 passivation layer which reduces nonspecific binding; (**b**) Normalized current versus time during cancer antigen 125 detection. When an antigen binds, the current decreases. The detection limit of 0.1 U/mL corresponds to 10 U/mL in the original serum, well below the 100–275 U/mL clinical levels used for cancer diagnosis. Adapted with permission from reference [24] (**c**) 2010 American Chemical Society.

Implementing biomaterials in electrical sensors has also improved sensor's performance by reducing nonspecific binding. Nonspecific binding of molecules to the surface of electrical sensors can significantly interfere with electrical sensor's performance, especially in metal nanowire and carbon nanotube devices which have high surface areas. One approach to overcome this issue is attaching analyte-specific receptors as well as a passivation layer to help reduce nonspecific binding. Recently, Chang *et al.* demonstrated how effectively functionalizing an In_2_O_3_ nanowire sensor with biomaterials and surface passivation suppresses the nonspecific binding signal and enables the sensor to achieve 1nM detection limits in both buffer and serum [24]. When molecules bind to the nanowire sensor, the electrical current passing through the nanowire will change and can be detected. However, nonspecific binding can also occur and cause changes, therefore limiting the sensitivity of the nanowire device. To enable specific detection, analyte-specific antibodies are attached to the nanowire, along with a passivation layer of Tween20 to block nonspecific binding. Then, using a built-in filtration system, whole blood samples from a finger prick are filtered and introduced to the nanowire sensor. These enhancements enable the nanowire sensor to detect cancer markers in whole blood at concentrations below clinically relevant levels (Figure 9). This device's cost-effective, rapid, and label-free detection capabilities could be used to develop highly efficient portable sensing devices.

Recent work on carbon nanotube-based FET sensors has demonstrated additional passivation approaches. Instead of attaching surface chemistry directly to the FET carbon nanotube sensor itself, an insulating graphene oxide membrane can be applied on top of the carbon nanotube sensor to act as a passivation layer [25]. The graphene oxide membrane can be subsequently functionalized with receptors for the analytes of interest. In addition to reducing nonspecific binding and possible degradation of the carbon nanotube sensors, the graphene oxide membrane improves the sensitivity and selectivity of the devices by improving the on/off ratio.

#### 3.3.3. Additional Considerations for Hybrid Electrical Sensors

By combining biological or synthetic materials with electrical sensors, the sensing capabilities, including sensitivity, signal to noise, and specificity, can be significantly improved. At the same time, introducing hybrid materials typically degrades the electrical performance because most hybrid layers have reduced conductivity as compared to the underlying sensor device. Also, the hybrid materials need to be robust so that they do not degrade while the electrical sensor is in operation. Therefore, it is critical to balance the potential improvement in sensor device performance with the inherent decrease in electrical device behavior.

In addition, consideration should be given to the compatibility of the hybrid materials with microfabrication processes. One of the significant advantages of electrical sensors over mechanical or optical sensors is their ease of integration with other on-chip components allowing multiplexing of sensors. However, most functional materials used in creating a hybrid device cannot tolerate high temperatures and harsh chemical environments and therefore, need to be added at the end of the fabrication process. In this case, the addition of the hybrid materials must be carefully optimized so that the materials and processes do not interfere with or damage the rest of the device.

## 4. Conclusions and Future Outlook

Over the past few decades, researchers have focused on developing sensors with ultra-low limits of detection. As a result, a wide range of devices with different operational modalities have been demonstrated. However, to continue to push the operating performance, other characteristics will need to be addressed, such as specificity and device lifetime. In order to transition these sensors out of a laboratory setting, other factors such as cost and robustness must also be considered. By combining optical, electrical, and mechanical-based sensing devices with new materials designed for biological and chemical detection, further advances in performance will be possible.

Recently, significant research has been invested in the development of organic and inorganic polymers for anti-biofouling applications and for encapsulation of solar cells. Many of these polymers would be ideally suited for biodetection applications, and would require minimal investment for the technology development. For example, the polysaccharide chitosan has been implemented as a biocompatible layer in various sensing applications. Adding a chitosan layer to MgO nanoparticles has been shown to minimize the nanoparticles' cytotoxic effects. In addition, by attaching complementary DNA strands to the MgO nanoparticles, single stranded DNA sequences could be detected at concentrations as low as ∼35 ng/μL with a 3–4 s response time. The sensors remained stable for 3–4 months when refrigerated [288]. In another application, a monolayer of 3-aminopropyl trimethoxysilane (APTMS) was applied to the oxide sensing layer of a FET biosensor. The APTMS modification decreases the work function by 2 eV and increases the threshold voltage by over 10 V, enabling electron affinity effects and field effects to be distinguished [289].

Additionally, numerous approaches are emerging for creating chemically and thermally stable binding sites. For example, using a technique based on replica molding called molecular imprinting polymers (MIP); it is possible to create antibody mimics. These binding sites are environmentally robust, allowing airborne pathogen detection. Alternatively, using library screening methods in combination with in silico design algorithms, it is possible to design and synthesize binding sites which are stable in specific environments.

However, many of the materials which will enable the next generation of sensors have yet to be developed or even envisioned. Just as conducting polymers enabled the field organic photovoltaics and OLEDs (organic light-emitting diodes); it is quite possible that material coatings could play a more active role in the sensor performance in the near future. For example, one could imagine a material coating which actively scavenges the environment, much like a jellyfish's tentacles, improving the surface area and collection efficiency of the device, or a material which actively filters a sample, much like a liver or kidney. Although these types of biologically-inspired materials have yet to be developed, their implementation will significantly improve a sensor's performance and reduce the complexity of any analysis system. As such, collaborations between materials researchers and sensor engineers will become increasingly important over the next decade.

## Figures and Tables

**Table 1.** Summary of different sensors, detection mechanism, and examples of detection. Additional details on each detection modality are found in the subsequent sections.

	**Electrical [3,5,17–53]**	**Mechanical [54–111]**	**Optical [1,9,14–16,112–137]**

	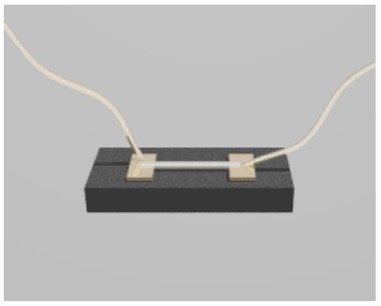	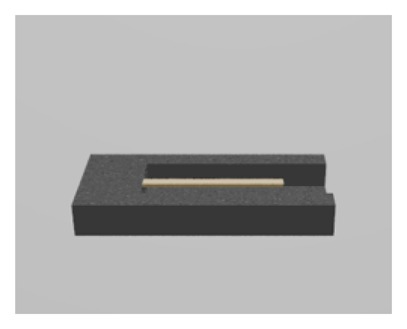	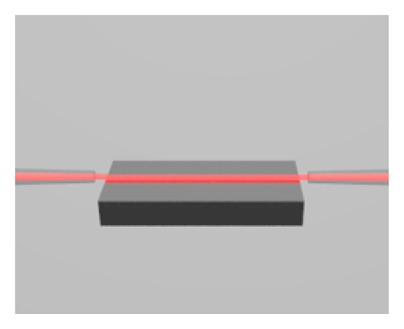
Detection approach	Change in: current, resistance, capacitance	Change in: stress, strain, vibrational frequency	Change in: power, refractive index, spectral signature
Materials	Silicon, carbon nanotubes, graphene, zinc oxide, indium phosphide	Silicon, carbon nanotubes, silicon nitride	Silica, silicon nitride, polymer, silicon
Types of experiments	Gas (e.g., CO_2_, N_2_), virus, protein (e.g., prostate specific antigen), bacteria	Gas (e.g., explosive vapors, solvents), virus, protein (e.g., prostate specific antigen), bacteria	Gas (e.g., humidity, solvents) virus, protein (e.g., prostate specific antigen), bacteria
